# “Mine Works Better”: Examining the Influence of Embodiment in Virtual Reality on the Sense of Agency During a Binary Motor Imagery Task With a Brain-Computer Interface

**DOI:** 10.3389/fpsyg.2021.806424

**Published:** 2021-12-24

**Authors:** Hamzah Ziadeh, David Gulyas, Louise Dørr Nielsen, Steffen Lehmann, Thomas Bendix Nielsen, Thomas Kim Kroman Kjeldsen, Bastian Ilsø Hougaard, Mads Jochumsen, Hendrik Knoche

**Affiliations:** ^1^Human Machine Interaction Lab, Department of Architecture, Design, and Media Technology, Institute for Architecture and Media Technology, Aalborg University, Aalborg, Denmark; ^2^Department of Health Science and Technology, Aalborg University, Aalborg, Denmark

**Keywords:** brain-computer interface (BCI), virtual reality, embodiment, agency, frustration, interaction paradigm

## Abstract

Motor imagery-based brain-computer interfaces (MI-BCI) have been proposed as a means for stroke rehabilitation, which combined with virtual reality allows for introducing game-based interactions into rehabilitation. However, the control of the MI-BCI may be difficult to obtain and users may face poor performance which frustrates them and potentially affects their motivation to use the technology. Decreases in motivation could be reduced by increasing the users' sense of agency over the system. The aim of this study was to understand whether embodiment (ownership) of a hand depicted in virtual reality can enhance the sense of agency to reduce frustration in an MI-BCI task. Twenty-two healthy participants participated in a within-subject study where their sense of agency was compared in two different embodiment experiences: 1) avatar hand (with body), or 2) abstract blocks. Both representations closed with a similar motion for spatial congruency and popped a balloon as a result. The hand/blocks were controlled through an online MI-BCI. Each condition consisted of 30 trials of MI-activation of the avatar hand/blocks. After each condition a questionnaire probed the participants' sense of agency, ownership, and frustration. Afterwards, a semi-structured interview was performed where the participants elaborated on their ratings. Both conditions supported similar levels of MI-BCI performance. A significant correlation between ownership and agency was observed (*r* = 0.47, *p* = 0.001). As intended, the avatar hand yielded much higher ownership than the blocks. When controlling for performance, ownership increased sense of agency. In conclusion, designers of BCI-based rehabilitation applications can draw on anthropomorphic avatars for the visual mapping of the trained limb to improve ownership. While not While not reducing frustration ownership can improve perceived agency given sufficient BCI performance. In future studies the findings should be validated in stroke patients since they may perceive agency and ownership differently than able-bodied users.

## 1. Introduction

Strokes are one of the leading causes of acquired disability in adults, but traditional rehabilitation techniques are not working properly for many patients due to the heterogeneity of the injury (Langhorne et al., 2009). Therefore, new rehabilitation techniques such as brain-computer interfaces (BCIs) incorporate motor learning principles to improve motor recovery after stroke (Krakauer, 2006; Grosse-Wentrup et al., 2011; Cervera et al., 2018). To regain control over ones body, BCI-based stroke rehabilitation aims at inducing neural plasticity, the proposed mechanism for skill acquisition (Pascual-Leone et al., 1995), in the affected brain areas, through activating motor cortical activity in them (Pichiorri et al., 2015). Activation of motor cortical activity can be obtained through motor imagery (MI) which appears to activate the same brain areas as executed movements (De Vries and Mulder, 2007). MI-BCI with visual feedback is effective in stroke rehabilitation (Pichiorri et al., 2015), but systems can also provide congruent somatosensory feedback in response to MI (Jochumsen et al., 2021). This could be through either functional electrical stimulation of the affected muscles or movement of the affected limbs with an exoskeleton or rehabilitation robot. BCIs based on the latter approach have improved motor function in stroke patients in several studies (see e.g., Ramos-Murguialday et al., 2013; Ang et al., 2014; Frolov et al., 2017; Biasiucci et al., 2018). BCI training and stroke rehabilitation in general involve a lot of monotonous and repetitive movements and tasks. Stroke patients felt bored when evaluating their rehabilitation (Langan et al., 2018). In order to keep motivation to train, patients need to stay engaged by e.g., gamification in the training (de Castro-Cros et al., 2020). According to a recent review, gamification increased rehabilitation outcomes over visual-only feedback (Karamians et al., 2020). To apply gamification to BCI-based rehabilitation, games must be designed to account for the relatively slow and simple input BCIs provide compared to e.g., a mouse and a keyboard. BCIs based on MI generally have a limited bandwidth, yielding rather low recall rates; the fraction of correctly identified MI attempts, even for a 2-class classification problem (MI vs. idle activity). Studies have reported different levels of recall rates for stroke patients performing MI or attempted movements, but they were roughly in the range of 50–85% (Ang et al., 2011, 2015; Pichiorri et al., 2011, 2015; Jochumsen et al., 2015b,c; Frolov et al., 2017; Biasiucci et al., 2018). Low recall rates of BCI systems can be due to technical aspects such as the pre-processing and classification algorithms that are employed, but for MI-based BCIs they can also be due to difficulties in performing MI—a skill that has to be learned. A strong sense of ownership—the users' feeling that a virtual limb belongs to their body—can accelerate learning how to perform MI (Alimardani et al., 2016a). Enriched multimodal feedback (visual and auditory) improved BCI performance for novice users over visual-only feedback consisting of a moving bar (Sollfrank et al., 2016), but yielded no benefit for BCI users after three training sessions (Nijboer et al., 2008). Similarly, combining different types of visual feedback through dummy face expressions along with flashing targets improved MI performance compared to providing such feedback separately (Zapała et al., 2018). Moreover, realistic feedback in virtual reality (VR) led to a 5% higher BCI performance than visual feedback in the form of an abstract bar (Skola and Liarokapis, 2018), although BCI performance when receiving abstract (moving bar) and realistic (moving hand) feedback did not differ (Neuper et al., 2009). Lastly, a BCI-controlled VR task showed that congruent feedback in response to an avatar yielded higher MI classification accuracy compared to spatially incongruent feedback (Braun et al., 2016; Sanchez-Vives et al., 2021). VR provides novel opportunities to design BCI-based rehabilitation tasks as it allows for dissociating visual experiences from the constraints bodies impose (Kilteni et al., 2012). To do so most efficiently requires an understanding of how generating body ownership illusions toward virtual bodies can be used to increase their perceived agency or reduce frustration and how to measure these constructs. Low recall rates cause frustration as users do not feel in control of their BCI triggered activations, i.e., their perceived agency of causing changes in the world is low. Unfortunately, the frustration decreases recall rates further and potentially increases the risk of patients not wanting to train with BCI systems (Kögel et al., 2020).

Outside of technically improving BCI recall, attempts at increasing sense of agency or reducing frustration have tried harnessing ownership—the feeling that an object is part of ones body (Nierula and Sanchez-Vives, 2019) or inducing an illusion of control (Vlek et al., 2014; Bashford and Mehring, 2016). To increase sense of agency, studies employed biased or so-called sham feedback (Barbero and Grosse-Wentrup, 2010; Alimardani et al., 2014; Evans et al., 2015). By giving participants the impression of successfully triggering MI-BCI even when their BCI output did not warrant activations, their perceived performance of the BCI was better than the actual decoded MI-BCI patterns (Barbero and Grosse-Wentrup, 2010; Alimardani et al., 2014; Evans et al., 2015). So, people experienced agency even without any actual control over activations (Vlek et al., 2014), and therefore delays between their ignored MI attempts and their 80% successes from (sham) feedback could have differed up to 3 s (Bashford and Mehring, 2016). Such positively biased feedback helped participants improve low MI activation rates but it reduced the subsequent actual MI activation rates of users who previously achieved higher activation rates (Barbero and Grosse-Wentrup, 2010). Similarly, people experienced significantly higher sense of agency over the movements of a robotic avatar when 10% of sham feedback (with activation delays up to 2.75 s) was added (90%, 4.6/7) to a genuine activation rate (80%, 3.1/7) (Alimardani et al., 2016a). A larger number of studies (van de Laar et al., 2013; Fard and Grosse-Wentrup, 2014; Hougaard et al., 2021) showed that higher activation rates increased sense of agency when input attempts yielded discrete feedback. But sense of agency did not depend on activation rates when visual feedback of a hand closing was temporally congruent, i.e., continuously updating without delay, during MI-BCI attempts (Kjeldsen et al., 2021). Removing the temporal congruency by adding delay of one or more seconds reduced sense of agency in both discrete (Alimardani et al., 2016b) and continuous BCI control (Evans et al., 2015). However, despite delays between 1 and 4 s users' sense of agency increased from system-generated input providing the same feedback as genuine (true positive) activations (for activation rates at 50%) (Hougaard et al., 2021). Spatial incongruency between MI-BCI clenching attempts and the resulting cursor movements, i.e., in the opposite direction expected from training, reduced sense of agency tremendously from 84 to 34% (Evans et al., 2015). Also, spatially incongruent feedback reduced sense of agency significantly compared to congruent feedback (Braun et al., 2016). The mismatch did not affect sense of agency when users expected the spatial incongruency between avatar - a virtual representation of their body, and MI-BCI movements (Skola and Liarokapis, 2018; Skola et al., 2019). Studies have relied on implicit and explicit measures of sense of agency (Zopf et al., 2018). Intentional binding, i.e., judgments about how much time has passed between MI attempts and their delayed feedback, has served as a measure of implicit agency as people underestimate these delays when experiencing high agency. Similarly, implicit measures of ownership have relied e.g., on using the magnitude of electrodermal responses when participants were made believe that their virtual limb was exposed to 'physical' harm (Petkova Valeria, 2011; Alimardani et al., 2016b; Bashford and Mehring, 2016). Explicit agency and ownership measurements have relied on self-reported rating scales.

Typically, studies investigated ownership of artificial limbs through rubber hand illusions, which trick users into feeling ownership over an artificial hand spatially aligned with their body (Botvinick and Cohen, 1998; Perez-Marcos et al., 2009; Petkova Valeria, 2011; Maselli and Slater, 2013; Bashford and Mehring, 2016; Braun et al., 2016; Pyasik et al., 2020). The illusion requires the users to have one of their hands concealed but exposed to stimuli (e.g., tickled by a feather) while they watch the rubber hand undergo the same stimulus at the same time so the users attribute the proprioceptive sensation to what they see (Botvinick and Cohen, 1998; Perez-Marcos et al., 2009; Petkova Valeria, 2011; Bashford and Mehring, 2016). Some authors hypothesized that the sense of ownership over the rubber hand creates the illusion of agency as users who felt a strong sense of ownership also felt a high perceived agency and believed they could move the rubber hand if they desired to according to explicit measures (Botvinick and Cohen, 1998; Perez-Marcos et al., 2009; Bashford and Mehring, 2016). However, one study showed that users can have a strong sense of ownership over a rubber hand without feeling agency over its movements when they received incongruent or sham feedback after MI-BCI input (Braun et al., 2016).

While perceived agency activates different parts of the brain than ownership, these senses can be hard to distinguish for voluntary movements where the user attempts to provide input (Gallagher, 2000). In voluntary MI-BCI movements, users attribute most positive feedback to be a result of their MI attempt regardless of having actual control or not (Alimardani et al., 2016a; Bashford and Mehring, 2016). However, we can observe ownership over limbs independently of perceived agency in involuntary movements where users observe their limbs being moved (Shimada et al., 2005) or in the anarchic hand syndrome in which patients experience high ownership but low sense of agency over their hands (Sala, 1998).

Visual representations resembling a human body or body part increased ownership compared to more abstract representations in spatially congruent setups. People felt less embodied by a sphere (-30%) (Zopf et al., 2018) and metallic grippers (-50%) than a humanoid hand (Alimardani et al., 2016a). Therefore, most studies induced ownership by having users control an avatar (Nierula and Sanchez-Vives, 2019) the representation of which consisted of either a full virtual body (Alimardani et al., 2016a,b) or just the part used for MI, e.g., a hand (Bashford and Mehring, 2016; Zopf et al., 2018). Only one study found higher ownership for a lower fidelity avatar depiction (a 2D black and white projection of a hand) compared to a three-dimensional highly realistic rubber hand. However, the former gave the impression of some form of agency over the discrete movements of the hand while the latter did not (Bashford and Mehring, 2016). Most recent studies aiming at inducing ownership spatially aligned the avatar's position as seen on a screen or through a head-mounted display (HMD) in VR with the participants' position (Petkova Valeria, 2011; Alimardani et al., 2016a,b; Skola and Liarokapis, 2018; Skola et al., 2019; Pyasik et al., 2020) since seeing an avatar from a first person point of view (POV), induced ownership over the whole virtual body and provided more ownership than a third person POV (Petkova Valeria, 2011; Maselli and Slater, 2013). A first person POV through a screen reduced ownership by removing the three dimensional spatial alignment provided by VR (Petkova Valeria, 2011). Similarly, temporal misalignments reduced users' ownership of virtual limbs in both voluntary and involuntary movements (Shimada et al., 2005; Alimardani et al., 2016b; Zopf et al., 2018). Delays as small as 200 ms between proprioceptive and visual feedback from ones own involuntarily moved body parts reduced ownership (Shimada et al., 2005). The mismatch experienced from an input hand's proprioception (voluntary movements) and the delayed (100–400 ms) visual feedback from the virtual hands reduced the ownership over those representations compared to a no delay condition (-5%) (Zopf et al., 2018) or a BCI condition without proprioceptive feedback (Alimardani et al., 2016b).

Only very few studies have investigated how to increase sense of agency and ownership through embodying the user with an anthropomorphic avatar (Alimardani et al., 2016a; Braun et al., 2016; Skola and Liarokapis, 2018; Zopf et al., 2018). A projected hand induced higher levels of ownership than a sphere but only increased explicit and not implicit agency of controlling the two representation in Zopf's study relying on motion capture input (Zopf et al., 2018). In the context of MI-BCI, Alimardani's studies evaluated sense of ownership and agency. Despite a large reduction in both sense of ownership and agency when comparing a metallic gripper to a humanoid hand, Alimardani could not verify the effect of sense of ownership on agency due to confounding reductions in MI-BCI performance (Alimardani et al., 2016a). Lastly, Braun's study reported that sense of agency decreased when providing spatially incongruent feedback which had lower ownership compared to congruent feedback, there was a significant correlation between sense of ownership and agency (Braun et al., 2016). A larger number of studies did not manipulate sense of ownership or agency as independent variables but hypothesized about their effect on one another (Alimardani et al., 2016b; Bashford and Mehring, 2016; Kokkinara et al., 2016; Skola and Liarokapis, 2018; Skola et al., 2019) based on their studies—see Table 1. Some argued that sense of agency and ownership correlate and cannot be observed independently (Alimardani et al., 2016b; Kokkinara et al., 2016). For example, MI-BCI users felt ownership over a moving avatar as long as they had sense of agency over its movement despite the lack of matching proprioceptive feedback (Alimardani et al., 2016b; Kokkinara et al., 2016). Others argued for ownership modulating sense of agency (Skola and Liarokapis, 2018; Zopf et al., 2018), e.g., in a *post-hoc* sub-group analysis Skola et al. found higher sense of agency for people with high ownership (Skola and Liarokapis, 2018), but their follow-up study could not support this correlation (Skola et al., 2019). Similarly, Bashford found that sense of agency and ownership correlated in their 80% sham input control setup but could not argue for a causal relationship (Bashford and Mehring, 2016). In conclusion, no study has successfully manipulated ownership while controlling for BCI activation and temporal congruency to understand whether ownership modulates sense of agency.

**Table 1:** Overview of previous work by medium (View), agent representation, whether it included an avatar, motor imagery task and the conjectured relationship between sense of agency and ownership as a result of the study (arrows indicate which factor moderates the other).

**References**	**View**	**Virtual agent**	**Avatar**	**MI task**	**Feedback**	**Conjectured** **relationship**
Alimardani et al. (2016a)	HMD	Hand (hum+Rob)	Yes	Hand clench	Discrete	SoO ← SoA^*^
Alimardani et al. (2016b)	HMD	Human Hand	Yes	Hand clench	Discrete	SoO ↔ SoA
Bashford and Mehring (2016)	proj.	Hand	No	Hand clench	Discrete	SoO ↔ SoA
Evans et al. (2015)	displ.	Cross	No	hand clench	Continuous	SoO ? SoA
Skola and Liarokapis (2018)	VR	Hand	Yes	Lift+finger ext.+press	Discrete	SoO → SoA
Skola et al. (2019)	VR	Hand	Yes	Pull + Push	Discrete	SoO ? SoA
Zopf et al. (2018)	proj.	Hand+Sphere	No	3D hand movement	Continuous	SoO → SoA^*^
Braun et al. (2016)	POV^**^	Robotic hand	No	Wrist extension/flexion	Discrete	SoO → SoA

* *studies that directly investigated the impact of ownership on sense of agency*.

** *This study used a first person point view (POV) without any virtual agents, but with a real robotic 2 hand.*

Thus, this study aimed to investigate whether higher levels of ownership from a humanoid hand in VR can enhance the perceived agency users feel over than hand's movements during an online MI-BCI task. This study had an additional goal of exploring the possibility of reducing users' frustration during the MI-BCI task through embodying them in a virtual body. Based on quantitative and qualitative findings, this study provides new knowledge on the relationship between ownership and perceived agency in a gamified MI-BCI task.

## 2. Methods

**Figure 1:**
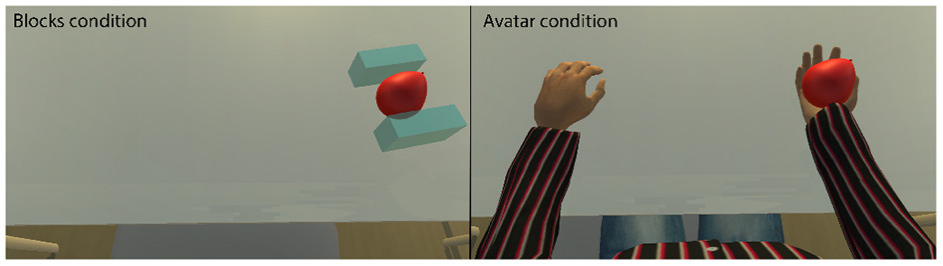
The different embodiment conditions in the design. In the avatar hand condition a hand held the balloon while two semi-transparent blocks surrounded the balloon in the blocks condition.

**Figure 2:**
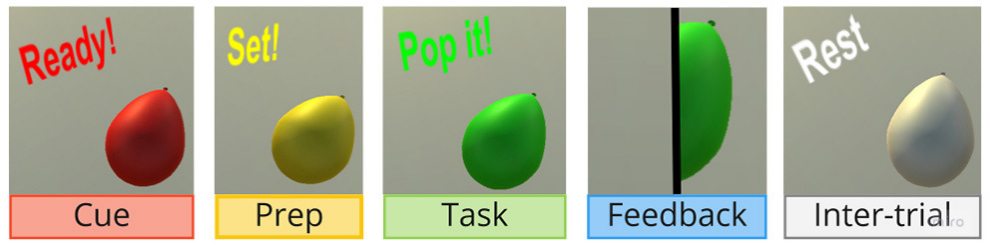
The visuals of the balloon that changed color during the five interaction phases in a traffic light style and then to white for the inter-trial period.

To understand the effect of ownership on sense of agency we created an interactive BCI-system using VR with two levels of embodiment (see Figure 1). The first level, embodying the users in a avatar hand aimed to induce a strong feeling of ownership whereas the second level, embodying the user in two blocks aimed to lower ownership in an interaction where balloons should be popped (see Figure 2). Both representations were spatially aligned with the palm of the participants actual hand during the experiment.

### 2.1. Participants

Twenty two able-bodied participants participated in the study (three female, nineteen male). All were university students ranging in ages between 20 and 28 (*M* = 24) years. Four previously tried a BCI interaction and had experience with MI while the rest had no experience.

In the absence of knowledge about effect sizes of our independent variables on perceived agency we relied on participant numbers from the most relevant previous studies (Braun and Clarke, 2006; Evans et al., 2015; Alimardani et al., 2016a,b; Bashford and Mehring, 2016; Skola and Liarokapis, 2018; Zopf et al., 2018; Skola et al., 2019) the median of which was 24. However, most of those studies followed between subject designs while our study used a within-subject design.

### 2.2. Procedure

**Figure 3:**
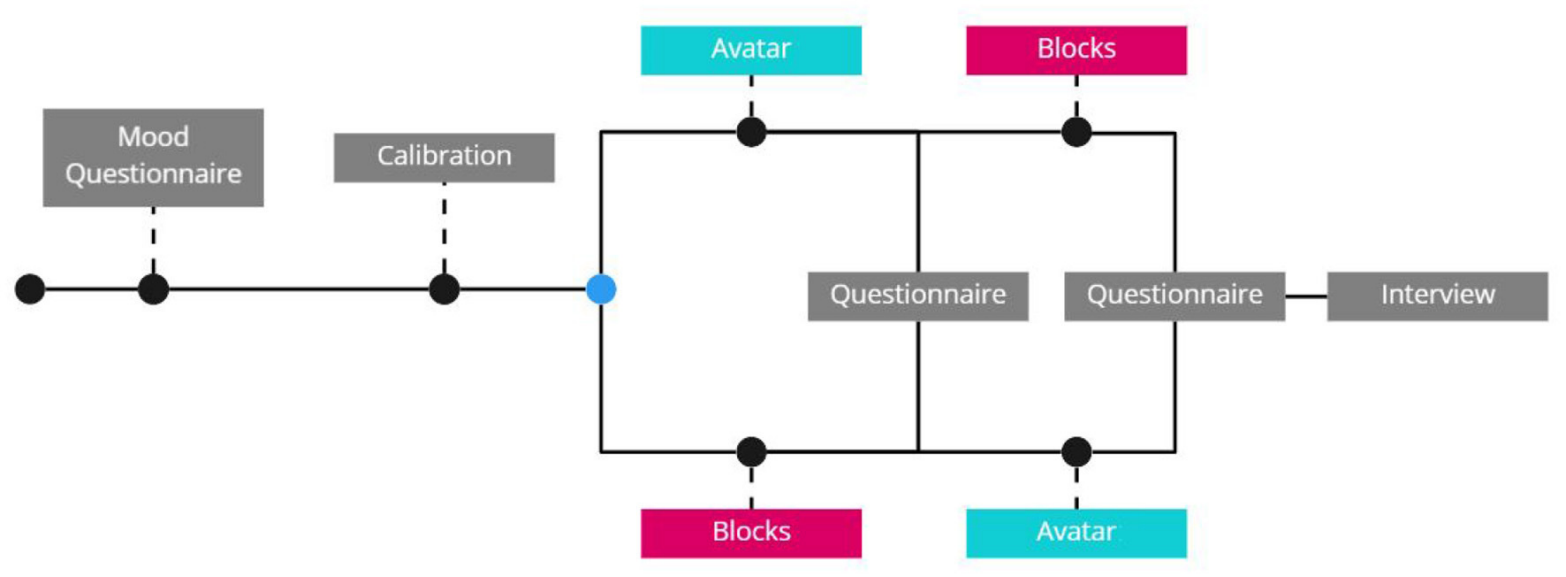
The procedure for the experiment started with the Mood questionnaire (Skola and Liarokapis, 2018), and equipping the EEG and VR headset. Afterwards the participants were allocated by order into each level and given a questionnaire after each condition followed by a debrief interview.

The participants read an instruction letter prior to the experiment. The instruction letter included a guide of the procedure, an explanation of how to perform MI and the calibration process, and a guide of the VR application used in the study without providing images of the avatar and blocks. After the participants finished an entry mood questionnaire a cap was mounted for recording the electroencephalography (EEG) and recorded kinesthetic MI for the BCI calibration (see Figure 3 for an overview of the experiment). Before the calibration process, a facilitator verbally explained how to perform kinesthetic MI again by imagining how the sensation of making a fist feels like in their hand. The participants clenched their fists to show that they have understood the movement and to gain reference for what to imagine. To calibrate the MI-BCI recognition, participants performed MI for 30 s in 30 trials when a cue appeared on a computer screen in front of them. To end the calibration, participants performed MI three more times while a facilitator monitored the BCI signal to verify that it can recognize their MI attempts. Then the participants donned a head-mounted display (Oculus Quest) through which they could see either an avatar with two hands that were spatially aligned with their body or two semi-transparent blocks (see Figure 1). The participants were seated in a chair with their hands resting on a table with their right hand palm facing up and left facing down. The experiment followed a within-subjects design and each condition (blocks, avatar hand), included 30 attempts of popping a balloon through MI attempts. After each condition, the participants answered a paper-based questionnaire measuring their perceived agency, ownership, frustration, and proprioception. The questionnaire allowed access to their previous ratings to guide a semi-structured debrief interview that concluded the experiment. During the interview, the participants explained how they comprehended the questions and elaborated on their ratings. We probed deeper into their experience during the task phase, how they felt about their performance, and what had affected their four ratings.

For the BCI calibration, the participants performed 30 kinesthetic MI attempts consisting of recalling the sensation of performing a palmar grasp of their right hand. For each attempt, the participants received a visual cue consisting of a red arrow pointing to the right and were instructed to maintain the imaginary contraction for 4 s. During this time period the participants were instructed to sit as still as possible, avoided blinking and activating any facial muscles. When the arrow cue disappeared, the participants rested. During the experiment, each MI attempt was preceded by a 5 s inter-trial pause, with the balloon in red and text offering to “feel free to look around.” During the cue phase, the text changed to red, saying *"Ready!"* for 3 s, see Figure 2. Afterwards, the balloon changed to yellow and the text to: *"Set!"* for 4 s. This signaled to the participant to prepare for performing MI. On the balloon turning green the text changed to: *"Pop it!"* to signify the task phase, which lasted for 5 s. Successful MI attempts during the task phase triggered an animation of the avatar hand clenching around the balloon or the blocks pressing against each other, see Figure 2. The balloon popped visually with matching audio feedback. Unsuccessful attempts yielded an alarming buzzer and the balloon changed color back to white while a new white balloon appeared in the same place for successful attempts. In either case this commenced the following inter-trial phase. The instruction letter explained all these phases with images as well as explaining that the balloon popped when the BCI recognizes their MI attempt and that a buzzer sound indicates a failed attempt.

### 2.3. Brain-Computer Interface

Using a Sintered EEG Electrode Cap (OpenBCI, USA) a Cyton biosensing board continuously recorded EEG signals from the following channels: F3, F4, C3, Cz, C4, P3, and P4 with respect to the international 10-20 System. The EEG was sampled at 250 HZ and grounded at AFz and referenced to CPz. The BCI used a modified version of the "Motor Imagery BCI" scenario from OpenViBE (Renard et al., 2010). There were only two modifications, 1) in the calibration of the BCI the arrow pointing to the left was substituted with the text “REST,” and 2) a detection was registered when the output of the classifier exceeded a participant-specific threshold for 0.5 consecutive seconds. The continuous EEG was bandpass filtered between 8 and 30 Hz with a 5th order Butterworth filter and a common spatial pattern filter, where the filter coefficients were derived from the calibration data. The common spatial pattern filter maximized the difference in the spectral power between the MI class and the rest class. The calibration data were divided into 1-s windows and this window shifted 1/16 s over the calibration data set. In each window the logarithmic band power was calculated from each electrode and used as an input feature for a linear discriminant analysis classifier. The decision boundary of the classifier was obtained through 5-fold cross-validation on the calibration data. In the online test, the BCI registered an MI event when eight (corresponding to 0.5 s, the refresh rate was 16 Hz) consecutive outputs of the classifier exceeded the participant-specific threshold. The requirement of the consecutive windows was added to make the detection of MI more conservative i.e., reduce the number of false positives. The threshold was selected based on the highest offline classification accuracy from the 5-fold cross-validation, but reduced with 20% to avoid it being too difficult to activate the BCI during the experiment. When the BCI detected an MI event during the experimental condition, the OpenViBE application sent the output to Unity through a TCP socket connection. The output of the classifier was logged during the task phases.

### 2.4. Measurements

For each condition, MI-BCI *performance* was calculated by dividing the number of successfully popped balloons by the total number of balloons (30). This assumed that participants attempted MI during each task phase. The debrief interview probed into whether participants tried not attempting to pop the balloon to understand potential false positives. None of the participants tried to do this.

The entry questionnaire drew heavily on Skola's (Skola et al., 2019) instrument and focused on a number of control variables including mood, motivation, and anxiety (a total of 14 Likert 7-point scale items) and two binary questions about previous BCI and VR experience. For each condition we obtained measures of sense of agency, ownership, proprioception, and frustration through 7-point Likert scales drawing on questionnaire items from Skola's and Alimardani's studies (Alimardani et al., 2016b; Skola and Liarokapis, 2018; Skola et al., 2019). The four items were:

- I think I was in control of the hands/blocks *(agency)*
- I felt like the hands/blocks were part of my own body *(ownership)*
- I could feel the movement of the virtual hands/blocks in my hand *(Proprioception)*
- I felt frustrated when trying to pop the balloon *(Frustration)*

### 2.5. Analysis

For comparisons between conditions we used Wilcoxon signed rank tests for self-reported measures and paired *t*-tests for BCI performance and multiple linear regressions for all other analyses. Neither their mood nor the order in which they experienced the conditions had a significant effect on sense of agency, ownership, proprioception, or frustration. None of the control variables nor prior experience with BCI or VR affected ownership, sense of agency, frustration, or BCI performance. Two of the authors independently coded disjoint parts of the debrief interviews and cross checked the resulting codes and themes together. After cross checking the independent codes, a thematic analysis synthesized these accounts (Braun and Clarke, 2006).

## 3. Results

**Figure 4:**
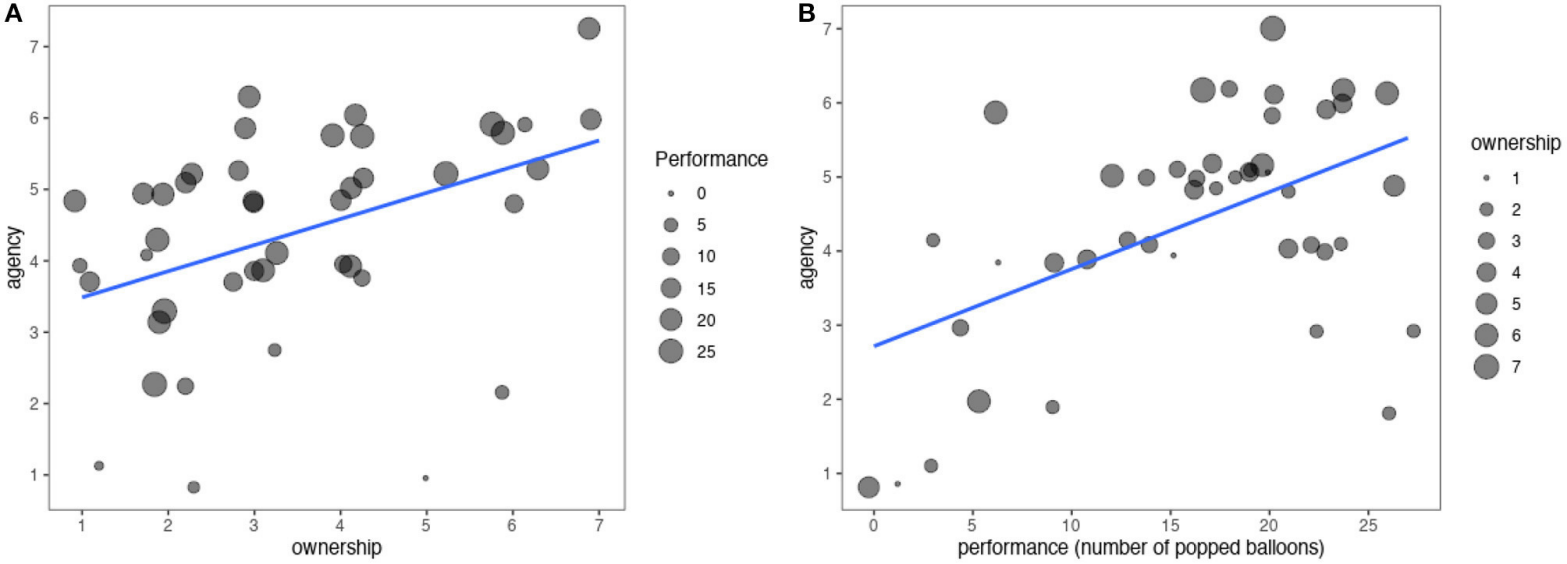
Perceived agency by ownership **(A)** and performance **(B)**, the blue lines represent the single factor linear fit. Categorical responses (ownership and agency) are jittered for better visibility.

From the percentage of popped balloons both the avatar hand (53%) and blocks (54%) condition supported similar levels of MI performance [*t*_(21)_ = −0.47, *p* = 0.6]. As intended, the avatar hand condition induced higher ownership (*V* = 194, *p* < 0.001) and proprioception levels (*V*=144, *p* < 0.001) than the blocks (see Table 2 for an overview). Motor imagery performance did not predict ownership ratings. The embodiment factor (avatar hand vs. blocks) did not affect sense of agency directly (*p* = 0.3) but the ownership across both conditions positively correlated with the perceived agency (*r*=0.47, *p*=0.001) and predicted it [*R*^2^=0.17, *F*_(1, 42)_=8.7, *p*=0.005]. However, ownership accounted only for 17% of the variance in sense of agency in Model 1. This was due to the confound from performance of popped balloons, which in a linear regression predicted 27% of the variance in sense of agency. A multiple regression showed that both ownership (β = 0.33, *p*=0.003) and performance (β=0.1, *p* < 0.001) significantly predicted sense of agency [*R*^2^=0.41, *F*_(2, 41)_=14.4, *p* < 0.001] and combined predicted 41% of the variance in sense of agency (see Figure 4). Higher ownership and higher performance both resulted in higher sense of agency. An introduced interaction effect between performance and embodiment in the regression model did not predict sense of agency and rendered all other predictors non-significant. Reported ownership did not moderate frustration directly but the sense of agency (β=−0.35, *p*=0.01) and performance (β=–0.1, p <0.001) predicted frustration a multiple regression [*R*^2^=0.51, *F*_(2, 41)_=21.1, *p* < 0.001]. Popping more balloons and experiencing higher agency reduced the participants' frustration. Users' reported proprioception correlated with performance in the avatar hand (*r* = 0.51, *p* = 0.014) but not the blocks condition (*p* = 0.8).

**Table 2:** Mean values and (standard deviation) per condition.

	**Ownership**	**Proprioception**	**Agency**	**Performance**	**Frustration**
Blocks	2.5 (1.0)	2.6 (1.4)	4.2 (1.3)	53% (25%)	3.6 (1.4)
Avatar	4.5 (1.6)	4.0 (1.7)	4.6 (1.6)	54% (25%)	3.7 (1.8)

In the debrief interviews, participants usually mentioned doing better in one condition than the other. As participants progressed, a majority of them (16) claimed having found a technique around 15 trials into the first condition to pop the balloon consistently. They described this as a particular thought or instinct they drew on which increased their perceived agency as they felt like they could control the BCI through their own intentions. However, failing a trial after finding a technique frustrated them and reduced their sense of agency as they felt they should not fail after learning to perform MI*"I did this 10 s ago, why can't I do it now?"* (P2).

Disentangling ownership from sense of agency proved difficult in the qualitative feedback as our participants often relied on accounts of both ownership and sense of agency to describe their experience. For example, *"I felt like the hands were a part of me so I was in control of what's going on"* (P5) and *"I felt disconnected from the body when it wouldn't move"* (P12). But almost all participants (16) felt more embodied in the avatar hand than the blocks condition. The visuals of an avatar sitting in the same position induced a sensation of ownership over the virtual body right after looking down at their body including the hand. Many (10) described making subtle adjustments to their seating and finger positions to match the avatar's hand to feel connected to it. The avatar hand movements matching the MI task added to the embodiment of 10 participants. *"The hand fit pretty well with what I imagined. The blocks felt alien to move like that"* (P4). However, when not successful in the hand condition this could cause frustration "*I felt betrayed by my own body* P(7)." While the objective performance data did not confirm it, many (17) expressed that controlling an avatar that embodied them made performing MI easier. They explained that seeing a visual representation of their hand performing the imaginary movement helped them picture the movement as well as how it felt like in their real hand. Since the instinct of clenching their hands came natural to them, a majority (11) reported feeling their muscles involuntarily twitch, tense, and activate. They felt their mental effort activating 'their' right hands, further strengthening their ownership over the avatar. Most participants (17) needing more mental effort to perform MI with the blocks because the blocks provided no reference for what they should imagine.

Controlling the avatar's hand seemed natural and straightforward like controlling their real hand to many (11) and induced strong sense of agency. Perceiving agency stemmed from both being embodied in the VR scene and executing the MI task. *"I felt the hands were a part of me so I was more in control of what was going on"* (P7). However, when they failed, the participants felt their control decreasing, as they could no longer control the avatar hand and became frustrated. For many (9) participants loss of control felt worse in the hand condition: *"It feels like I've lost control of my body"* (P7) and *"The blocks are arbitrary, they don't matter. But the hands feel like a part of me so it didn't feel right"* (P8). A few (5) complained that the avatar did not react to their MI fast enough. Since they felt the body belonged to them, they felt like the avatar should have popped the balloon almost as soon as they thought of it *"I thought it would have come more naturally since it was my hand I was looking at. The other one was just two blocks but it was the same"* (P10). In the blocks condition, participants saw the blocks as objects that they controlled rather than a representation of them. To many (9), clenching the avatar's hand felt like an action they performed, while the blocks' movement resembled feedback for doing the task correctly. Many (13) participants felt frustrated from the reduced sense of agency when failing to pop the balloon. The participants blamed themselves as they felt incapable of controlling their brain activity *"When you're sitting there trying really hard to make the hand move and then it doesn't move an inch, you feel responsible for that and it frustrated me"* (P9).

**Figure 5:**
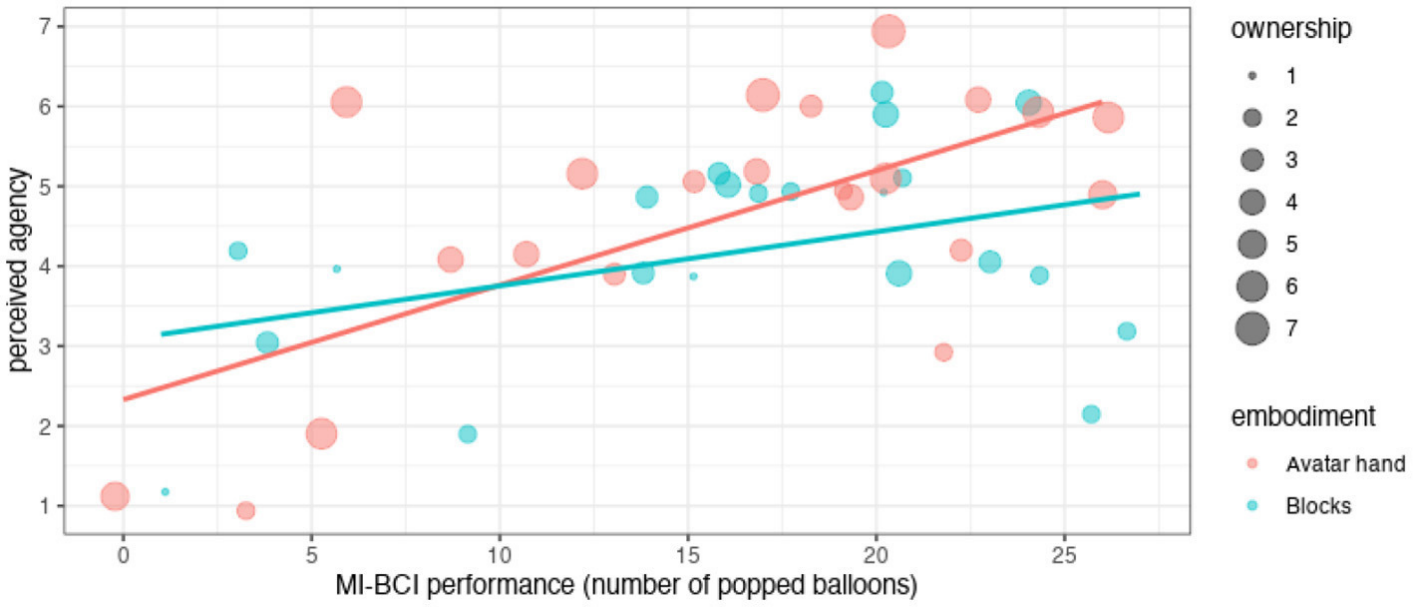
Perceived agency depending on performance and ownership by condition.

A follow-up analysis lend support for the qualitative results. Specifically, we tested whether adding an interaction effect between embodiment and performance to Model 2 predicted perceived agency better. The multiple regression model showed that ownership (β = 0.41, *p*=0.003) and an interaction effect (β = 0.09, *p*=0.064, for avatar hand condition) between performance (β = 0.05, *p*=0.13) and embodiment (β=–1.79, *p*=0.051, β for avatar hand condition) were significant or close to significant predictors of perceived agency [*R*^2^=0.47, *F*_(4, 39)_=8.6, *p* < 0.001]. See Figure 5 for an illustration of the interaction effect. Combined they predicted 47% of the variance in perceived agency (Model 3). While the interaction effect between embodiment and performance only bordered significance—potentially due to insufficient statistical power—these result matched the qualitative feedback.

## 4. Discussion

Our study succeeded in modulating ownership to a large degree by contrasting am avatar's hand with abstract blocks both of which provided spatially and temporally congruent feedback. Our findings ran counter to previous suggestions that perceived agency and ownership cannot be manipulated independently (Kilteni et al., 2012; Bashford and Mehring, 2016; Kokkinara et al., 2016; Zopf et al., 2018) but aligned with Skola's and Pyasik's findings (Skola and Liarokapis, 2018; Pyasik et al., 2021). Participants in both our and Skola's study achieved similar levels of MI performance (in the order of 55%). But, while Skola (Skola and Liarokapis, 2018) found no correlation between ownership and perceived agency, our study found a significant effect of ownership on perceived agency. However, Skola's task lacked congruency between the MI task (palmar grasp) and the resulting avatar movements (hand raising), which our study aligned completely and many participants referred to. Similarly, spatial congruency increased sense of agency in a VR-based MI-BCI study that kept ownership constant in a similar setup to ours (Kjeldsen et al., 2021). Conversely, a number of participants complained about the confusion and distraction Skola's feedback caused. Our results provide evidence for Alimardani's (Alimardani et al., 2016a) hypothesis that ownership modulates sense of agency. However, we observed no effect of the embodiment factor (hand vs. blocks) on sense of agency while Alimardani who found a large effect size from their embodied conditions (hand vs. a gripper, Cohen's *d*=1.42)—most likely due to their large unaccounted differences in performance between conditions and feedback incongruency for the gripper. While our study provided feedback congruent to the MI task in both conditions and controlled for performance.

Our initial results were inconclusive on why the embodiment factor itself did not affect sense of agency but only the resulting ownership did—even when controlling for performance. Our qualitative feedback hinted at participants holding the avatar hand to a higher standard than the blocks and potentially penalizing perceived agency when performance was low, which, considering the large spread of BCI performance (see Figure 4), happened for many participants. The follow-up analysis confirmed this as a plausible explanation. This disappointment could also serve as an explanation for why higher ownership did not reduce frustration while increasing perceived agency. An insufficient statistical power to measure significant differences in frustration from ownership due to the high variance in performance and the large effect of performance on both perceived agency and frustration provides an alternative or complementary explanation. The latter explanation matches the qualitative accounts in which participants often justified their frustration ratings with a lack of performance rather than not feeling ownership. Taken together this suggests that performance impacts agency and frustration separately from ownership, which remained unaltered by low performance. This supports our finding that ownership and agency can be manipulated independently. In contrast, Alimardani et al. (2016a,b) and Bashford and Mehring (2016) posited that ownership correlates with agency, and thus decreases when performance falls below 70% BCI performance. Both studies could not conclude on their hypothesis as they held BCI performance at a 70% level. But so far no study including ours has provided evidence to the link between ownership and performance. This includes studies in which participants achieved BCI performance levels between 0 and 100% in the different conditions with a 70% average (Kjeldsen et al., 2021). On the contrary, our participants achieved lower BCI performance (on average 54%) with the spread of ownership mirroring those of previous studies (Alimardani et al., 2016b; Skola and Liarokapis, 2018; Kjeldsen et al., 2021).

It should be noted that the BCI performance in this study (number of true positives) is heavily influenced by the way the MI detector is constructed in the BCI. By having the output of the classifier exceed the threshold for eight consecutive windows (i.e., 0.5 s) reduce the number of activations due to noise (false positives), but the trade-off is a lower number of true positive detections. This increases the likelihood that the detections that were observed in this study were actually true positives rather than false positives. Since there is no access to the ground truth it is not possible to know whether it was a false positive or a true positive. In future studies, a small test run of the BCI could be performed before/after the actual intervention to estimate the number of false positives/negatives and true positives. Here the participant would need to verbally indicate if he/she intended to perform MI (Jochumsen et al., 2015a), or perform the MI at precise time stamps (e.g., within ± 1 s).

## 5. Conclusion

Anthropomorphic feedback in line with an MI task from a first person POV providing spatially and temporally aligned motor congruency can increase ownership in MI BCI systems. MI BCI performance explains most of the variation in both sense of agency and frustration. When controlling for BCI performance higher ownership over virtual limbs does not reduce frustration but increases sense of agency. Designers of MI-BCI systems should draw on additional means to reduce frustration e.g., fabricated input (Hougaard et al., 2021). Given the positive effect on ownership and indirectly agency anthropomorphic representations can be recommended for MI-BCI training tasks unless BCI performance is low. Our qualitative data and thematic analysis provided a useful lens into the participants' experiences—a method currently underutilized in BCI studies. Future studies should validate our findings with stroke patients who may experience sense of agency and ownership differently given their injury. As indicated by Kögel et al. (2020), motor-impaired patients feel weaker sense of agency during BCI training as they feel the lack of control with the BCI is similar to their paralysis.

## Data Availability Statement

The original contributions presented in the study are included in the article/supplementary material, further inquiries can be directed to the corresponding author.

## Ethics Statement

Ethical review and approval was not required for the study on human participants in accordance with the local legislation and institutional requirements. The patients/participants provided their written informed consent to participate in this study.

## Author Contributions

HZ, DG, LN, TN, TK, and SL: conceived and designed the study, recruited participants, conducted study, collected and performed the statistical analysis, wrote first draft of manuscript, and revised the submitted version. HK, BH, MJ, and HZ: wrote, revised, and edited the submitted manuscript. HK and BH: supervised project, revised and guided the design of the study and performed statistical analysis, and revised and edited the first manuscript draft. All authors contributed to the article and approved the submitted version.

## Funding

This research was partially funded by VELUX FONDEN (project number 22357).

## Conflict of Interest

The authors declare that the research was conducted in the absence of any commercial or financial relationships that could be construed as a potential conflict of interest.

## Publisher's Note

All claims expressed in this article are solely those of the authors and do not necessarily represent those of their affiliated organizations, or those of the publisher, the editors and the reviewers. Any product that may be evaluated in this article, or claim that may be made by its manufacturer, is not guaranteed or endorsed by the publisher.
